# Evaluation and Optimization of Quality Based on the Physicochemical Characteristics and Metabolites Changes of Qingpi during Storage

**DOI:** 10.3390/foods12030463

**Published:** 2023-01-19

**Authors:** Yunxia Cheng, Cui Wu, Zhenying Liu, Pingping Song, Bo Xu, Zhimao Chao

**Affiliations:** Institute of Chinese Materia Medica, China Academy of Chinese Medical Sciences, Beijing 100700, China

**Keywords:** Qingpi, storage years, quality changes, physicochemical characteristics, color, flavonoids, antioxidant activity, metabolites

## Abstract

Qingpi, the dried immature pericarp of *Citrus reticulata* Blanco, is a commonly used medicinal food with some health-promoting benefits. In general, it is essential that Qingpi be stored for a period of time, but there are no reports about the number of storage years needed to obtain the best quality of Qingpi. Our aim was to determine the best storage time of Qingpi by studying the physicochemical properties and metabolite changes in product stored from 1 to 5 years. As a result, the color of Qingpi became darker during storage. Both the levels of three flavonoids (hesperidin, nobiletin, and tangeretin) and total flavonoids (TFs) and the antioxidant activity decreased during storage and the total phenolics (TPs) content fluctuated during storage. Cluster analysis was performed on the color parameters measured using a color difference meter, revealing that the color of Qingpi differed before and after 3 years of storage. A total of 9 special differential metabolites were identified that could be used to distinguish the storage years of Qingpi. This is the first study to report the quality changes of Qingpi during storage. The optimized results of the quality evaluation indicated that Qingpi should be stored for no more than 3 years.

## 1. Introduction

*Citrus reticulata* Blanco (family Rutaceae) is a fruit tree widely distributed all over the world. In the Xinhui District, Guangdong Province, the immature pericarp of *Citrus reticulata* ‘Chachi’ (cultivar of *C. reticulata*) is peeled off and dried under sunlight from July to August each year in order to obtain the medicinal and edible Qingpi [1,2]. Qingpi, a commonly used traditional Chinese Medicine, is used to treat distention and pain in both the chest and hypochondriac region, hernia pain, mass formation in the breast, mastitis, and abdominal pain due to the retention of undigested food [3].

Qingpi is rich in phenolic compounds, especially flavonoids. Flavonoids have many beneficial effects on human health and can be divided into flavanone glycosides (such as hesperidin and naringin) and polymethoxylated flavones (such as nobiletin and tangeretin) [4,5]. Modern studies have found that some flavonoids, such as narirutin, nobiletin, and tangeretin, can reduce liver damage and prevent liver disease [2]. Hesperidin has anti-oxidant, anti-inflammatory, neuroprotective, and anti-tumor activities [5]. Naringin possesses strong anti-oxidant, anti-inflammatory, and anti-cancer activities and has potential preventative effects against chronic colorectal inflammation and cancer [6,7]. Nobiletin can inhibit hepatic lipogenesis via activation of AMP-activated protein kinase [8], has anti-tumor and anti-inflammatory effects, and can prevent arterial thrombosis [9]. Tangeretin has anti-cancer, anti-inflammatory, anti-atherosclerosis, liver protection, and neuroprotective effects [10]. In addition, Qingpi is a statutory medicinal food and officially authorized by the National Health Commission of the People’s Republic of China [11,12]. Some studies about Qingpi have been published in food-related journals [1,13,14]. In terms of edible usages, Qingpi is a popular brewing material. It can be brewed alone in hot water, but also combined with different varieties of tea to prepare new tea drinks, such as Gan-Pu tea (together with Pu-er tea), Qingpi-black tea (together with black tea), Qingpi-flower tea (together with a variety of flower teas) [15,16,17,18]. The combination of tea and citrus pericarp as a brewed tea has a history of thousands of years [19,20]. Qingpi can also be used in a medicinal diet, including porridge, soup, and medicated wine [21]. With the transformation of medical purposes and health concepts, healthy foods with disease prevention benefits have become increasingly popular [22]. Qingpi, as a medicinal and edible material, meets people′s needs for healthy food and has attracted great interest among consumers in the healthcare industry.

Chenpi, dried mature pericarp of *C. reticulata*, has same origin as Qingpi, but has a different harvest time from September to December [1]. Chenpi needs to be stored for at least 3 years to obtain better quality, usually for 10 years or more [23,24]. The storage process is called “aging” [25]. According to our previous investigation of the quality of Qingpi in Xinhui District, Qingpi also needs to be stored for a period of time to produce a darker color, but a longer storage time does not necessarily result in better quality. At present, the quality changes of Qingpi during storage have not been reported. There are also no reports of the optimal number of storage years needed to obtain the best quality of Qingpi.

In this study, Qingpi samples from a same planting base were harvested in 5 consecutive years and stored under the same storage conditions for 5 to 1 years to obtain Qingpi 5 to Qingpi 1, respectively. In order to determine the best number of storage years, the physicochemical characteristics and metabolite changes of Qingpi samples stored for 1 to 5 years were studied. Among them, the physicochemical indexes included color, levels of three flavonoids (hesperidin, nobiletin, and tangeretin), total flavonoids (TFs) content, total phenolics (TPs) content, and antioxidant activity. These data were analyzed to obtain a reasonable storage time with the best quality. In addition, Qingpi samples with different storage years were distinguished.

## 2. Materials and Methods

### 2.1. Materials and Chemicals

#### 2.1.1. Materials

The fresh fruits of *C.* *reticulata* Blanco were harvested on a same day (20 July) of each year in 2020, 2019, 2018, 2017, and 2016 in the same plantation of the Changwen Professional Planting Base, which is located at 22°30′ north latitude and 112°56′ east longitude in Xinhui District, Guangdong Province, China. The plant was identified as *Citrus reticulata* ‘Chachi’ (family Rutaceae) by Prof. Zhimao Chao (Institute of Chinese Materia Medica, China Academy of Chinese Medical Sciences). The fresh immature pericarps were peeled off, dried under sunlight, placed in food grade plastic baskets, and stored for 1, 2, 3, 4, and 5 years in the same warehouse of Changwen Professional Planting Base to obtain samples Qingpi 1, Qingpi 2, Qingpi 3, Qingpi 4, and Qingpi 5, respectively. The temperature of the storeroom ranged from 10 to 40 °C and the relative humidity ranged from 40% to 85%. All samples of Qingpi were removed from storage 21 July 2021 for our experiment. The certificate specimens were deposited at Laboratory 1022 of the Institute of Chinese Materia Medica, China Academy of Chinese Medical Sciences, Beijing, China.

#### 2.1.2. Chemicals

HPLC-grade and MS-grade acetonitrile and methanol were purchased from Thermo Fisher (Osterode, Germany). MS-grade formic acid was obtained from CNW Technologies GmbH (Dusseldorf, Germany). 6-hydroxy-2,5,7,8-tetramethylchromane-2-carboxylic acid (Trolox), 2,4,6-tripyridyl-s-triazine (TPTZ), and Folin-Ciocalteu phenol reagent were obtained from Sigma Aldrich (St. Louis, MO, USA). 1,1-diphenyl-2-(2,4,6-trinitrophenyl) hydrazine (DPPH) was obtained from Shanghai Aladdin Bio-Chem Technology Co., Ltd. (Shanghai, China). Reference standards of hesperidin (AF8012603), narirutin (AF7112219), and gallic acid (AF8062803) were purchased from Alfa Biotechnology Co., Ltd. (Chengdu, China). Catechin (AF9051402), nobiletin (HH206560198), hesperetin (HH206898198), and tangeretin (HA061118198) were purchased from Chenguang Biotechnology Co., Ltd. (Baoji, China). Sodium nitrite (090612) was obtained from Beijing Hongxing Chemical Plant (Beijing, China). Phosphoric acid (20120100), acetic acid (20050902), and sodium hydroxide (20111012) were obtained from Beijing Chemical Plant (Beijing, China). Aluminum nitrate (20181017) and sodium acetate trihydrate (20190602) were purchased from Fu Chen Chemical Reagent Co., Ltd. (Tianjin, China). Sodium carbonate (C10855106) was obtained from Shanghai Macklin Biochemical Co., Ltd. (Shanghai, China). Ferric chloride (20194032093) was obtained from Tianjin Zhiyuan Chemical Reagent Co., Ltd. (Tianjin, China). Ultrapure water was produced using a Milli-Q water purification system (Millipore, Billerica, MA, USA).

### 2.2. Color Evaluation

A DFT-50A pulverizer (Linda Machinery Co. Ltd., Hangzhou, China) was used to crush the Qingpi samples into powder (65 mesh). The color evaluation was performed using an NH310 color difference meter (Shenzhen Three NH Technology Co. Ltd., Shenzhen, China) and values of the color parameters L*, a*, b*, c*, and h* were obtained. The L*, a*, and b* values represented lightness, greenness (negative) to redness (positive), and blueness (negative) to yellowness (positive), respectively. The c* value defined the saturation of color and the h^*^ value indicated the difference between color and gray [26]. The color difference meter was calibrated based on the white tile standard of the instrument. A powder sample of 3.5 g was put into the test box and measured using the colorimeter. Samples were analyzed three times in triplicate experiments, and the results were expressed as mean ± standard deviation. The total color difference (ΔE*) was calculated by the following equation:

$$\Delta E^{*}=\sqrt{{\left(L^{*}-L_{0}^{*}\right)}^{2}+{\left(a^{*}-a_{0}^{*}\right)}^{2}+{\left(b^{*}-b_{0}^{*}\right)}^{2}}$$

where *L*_0_*, *a*_0_*, and *b*_0_* were the values of the Qingpi 1 sample.

### 2.3. Flavonoids Analysis with HPLC

#### 2.3.1. HPLC Conditions

The flavonoids were detected by the diode array detector (DAD) using a Shimadzu LC-20AT HPLC system (Shimadzu Corporation, Tokyo, Japan). The system was equipped with a CBM-20A system controller (Shimadzu Corporation, Tokyo, Japan), an LC-20AT pump (Shimadzu Corporation, Tokyo, Japan), a CTD-10ASvp column oven (Shimadzu Corporation, Tokyo, Japan), an SPD-M20A UV-vis detector (Shimadzu Corporation, Tokyo, Japan), a SIL-20A auto injector (Shimadzu Corporation, Tokyo, Japan), a DGU-20A_5_ degasser (Shimadzu Corporation, Tokyo, Japan), and a Shimadzu LC-solution workstation (Shimadzu Corporation, Tokyo, Japan). Compounds were separated using an Agilent ZORBAX SB C_18_ column (4.6 mm × 250 mm, 5 μm). The solvent system consisted of acetonitrile (A) and water (B). The gradient elution program was as follows: 19% A for 0–10 min, 19–50% A for 10–25 min, and 50–56% A for 25–40 min. The flow rate was 1.0 mL/min. The column temperature was kept at 35 °C. The injection volume was 10 μL. The effluents were monitored based on the maximum absorption wavelength at 284 nm for hesperidin and 330 nm for nobiletin and tangeretin.

#### 2.3.2. Preparation of Sample Solutions

Qingpi samples were crushed into powder (65 mesh) using a pulverizer. A powder sample of 0.2 g was accurately weighed, put into 50 mL stoppered conical flask, and 30 mL of methanol was added. The flask was weighed using a CP224C electronic balance (Ohaus Instruments Co. Ltd., Shanghai, China), the sample was extracted for 50 min using a KQ-100E ultrasonicator (Kunshan Ultrasonic Instruments Co. Ltd., Kunshan, China), and the flask was weighed again after being cooled. The missing weight after sonication of the extract was made up with methanol, mixed well, and the sample was filtered through filter paper. The obtained filtrate was filtered again through a 0.45 μm membrane filter before HPLC analysis.

#### 2.3.3. Preparation of Standard Solutions

A mixed standard stock solution was prepared by dissolving the reference compounds of hesperidin, nobiletin, and tangeretin with methanol. The final concentrations were 201.0, 25.68, and 15.68 mg/mL [3], respectively. The standard solution was filtered through a 0.45 μm membrane filter before HPLC analysis.

#### 2.3.4. Establishment of Calibration Curves

The mixed standard stock solution was gradually diluted with methanol to obtain six standard solutions with different concentrations. All standard solutions were analyzed by the described HPLC conditions. A linear relationship for each compound was established by drawing peak areas (*Y*) against the corresponding concentration of the standard solutions (*X*, mg/mL) in the range of detection [23].

#### 2.3.5. Method Validation

Method validation was evaluated for precision, stability, repeatability, and recovery. The precision of the HPLC method was estimated by six consecutive injections of a standard solution. The stability of the sample solution was assessed by injecting a sample solution that was stored at room temperature (23 °C) for 0, 4, 8, 12, 24, 36, and 48 h. The repeatability of the HPLC method was determined based on six replicate analyses from the same sample. A known amount of mixed standard solution was added to the sample (0.25 g) that was previously quantified, and the spiked sample was analyzed using the described method to determine the recovery rate.

### 2.4. Determination of Total Flavonoids (TFs)

The TF content in Qingpi samples was measured using a colorimetric assay [27]. A 4.0 mL aliquot of sample solution (obtained in Section 2.3.2) was mixed with 1.0 mL 5% NaNO_2_ solution and 6.0 mL ultrapure water in a brown flask and shaken well. The mixed solution was allowed to stand for 6 min at room temperature (23 °C). A 1.0 mL volume of 10% Al(NO_3_)_3_ solution was added to the flask, mixed well, and kept for 6 min at room temperature (23 °C). Then, 10.0 mL 1 M NaOH solution was added to the flask and mixed well. The final volume was maintained at 25 mL with ultrapure water. The absorbance of the solution at 510 nm was determined against that of a blank using a spectrophotometer. The results were expressed as mg of catechin equivalents (CE) per gram of dry weight (DW) for Qingpi (mg CE/g DW).

### 2.5. Determination of Total Phenolics (TPs)

The TP content in Qingpi samples was determined using a Folin-Ciocalteu colorimetric method with slight modification about the literature [27]. The sample solution (obtained in step Section 2.3.2) of 50 mL was mixed with 0.5 mL 2 M of Folin-Ciocalteu reagent and 6.0 mL ultrapure water in a brown flask. The mixed solution was allowed to stand for 4 min at room temperature (23 °C). A 1.5 mL volume of 20% Na_2_CO_3_ solution (*w*/*v*) was added to the mixture and the final volume was adjusted to 25 mL with ultrapure water. After incubating for 30 min in a water bath at 30 °C in the dark, the absorbance was determined at 765 nm against that of a blank using a TU-1810ASPC UV-visible spectrophotometer (Purkinje General Instrument Co. Ltd., Beijing, China). The results were expressed as mg of gallic acid equivalents (GAE) per gram DW for Qingpi (mg GAE/g DW).

### 2.6. Evaluation of Antioxidant Activity

The antioxidant activity of Qingpi was determined by DPPH method and ferric-reducing antioxidant potential (FRAP) method with Trolox as the reference using a UV-visible spectrophotometer.

The DPPH value was determined according to a published procedure with minor modifications [28]. A 0.1 mL aliquot of the sample solution (obtained in Section 2.3.2) was mixed with 69.9 mM of DPPH methanol solution and the final volume was kept at 5.0 mL. The mixed solution was reacted for 30 min at room temperature (23 °C) in the dark and the absorbance was measured at 517 nm.

The FRAP value was determined according to a published method with slight modifications [28]. A 0.05 mL aliquot of the sample solution (obtained in Section 2.3.2) was mixed with 5.0 mL FRAP reagent containing 20 mmol/L FeCl_3_, 10 mmol/L TPTZ, 40 mmol/L HCl, and 0.3 mol/L acetate buffer (pH 3.6), reacted for 35 min at room temperature (23 °C) in the dark, and the absorbance was measured at 593 nm. The results of DPPH and FRAP were expressed as mM Trolox equivalents (TE) per gram DW for Qingpi (mM TE/g DW).

### 2.7. Untargeted Metabolomics Analysis of Qingpi

#### 2.7.1. Preparation of Experimental Solutions

The sample solution obtained in Section 2.3.2 was filtered through a 0.22 μm membrane filter, transferred to an LC vial, and stored at −80 °C for LC-MS analysis. The standards of hesperidin, nobiletin, tangeretin, hesperetin, and narirutin were dissolved with methanol to prepare a mixed standard solution. A quality control (QC) sample solution was prepared by mixing extract solutions of all Qingpi samples in equal volumes of 1 mL [29]. The volume of the QC sample solution was equal to that of every sample solution for LC-MS analysis.

#### 2.7.2. UPLC-MS Conditions

An ACQUITY I-class UPLC system equipped with a Xevo G2-XS Q-TOF MS instrument (Waters Corporation, Milford, MA, USA) was used to analyze the Qingpi samples. Compounds were separated using a Waters ACQUITY UPLC HSS T3 chromatographic column (2.1 mm × 100 mm, 1.8 μm; MA, USA). The injection volume was 4 μL and the flow rate was 0.4 mL/min. The temperature of column was set at 40 °C. The mobile phase was composed of water containing 0.1% (*v*/*v*) formic acid (A) and acetonitrile containing 0.1% (*v*/*v*) formic acid (B). The gradient elution program was as follows: 0–15 min for 99–1% A, 15–17 min for 1% A, 17–17.10 min for 1–99% A, and 17.10–20 min for 99% A.

For the MS conditions, the positive mode of electrospray ionization (ESI) source was used, the mass range was *m*/*z* 50 to 1200, and the scanning time was 0.25 s. The voltage was set at +2.5 KV for capillary, 40 V for cone hole voltage, 80 V for ion source compensation, and +3.0 KV for spray voltage. The cone hole gas flow rate was 50 L/h. The desolvation gas temperature was 450 °C and its flow rate was 800 L/h. The low collision energy was set as 6 eV and the high collision energy increased from 15 to 50 eV. The MS^E^ data acquisition mode was used to collect data in real time.

#### 2.7.3. Data Preprocessing and Analysis

The raw UPLC-QTOF-MS data were collected with Waters Masslynx4.1 software (Waters Corporation, Milford, MA, USA) and then imported into Progenesis QI v2.3 software (Nonlinear Dynamics, Newcastle, UK) for baseline filtering, peak identification, integration, retention time correction, peak alignment, peak extraction, and normalization [30]. Next, the MS data, including ANOVA *p* < 0.05, max fold change > 1.5, retention time, *m*/*z*, chromatographic peak width, and isotope distribution were derived.

The obtained MS data were imported into both Origin 2022 software (OriginLab Corporation, Northampton, MA, USA) and SIMCA-P software (14.1, Umetrics, Umeå, Sweden) for principal component analysis (PCA), partial least squares discriminant analysis (PLS-DA), and variable importance of projection (VIP). In this study, special differential metabolites were considered to meet the following identification conditions: ANOVA *p* < 0.05 and max fold change > 1.5 (labeled in Progenesis QI software) and VIP value > 2.0 (labeled in PLS-DA analysis results). In order to intuitively display the metabolite differences and achieve rapid discrimination of samples with different storage years, the MS data of the identified differential metabolites were imported into the Metabo Analyst 5.0 website to plot a heat map.

### 2.8. Statistical Analysis

Samples were analyzed three times in triplicate experiments. The obtained results were expressed as means ± standard deviation (SD). Hierarchical cluster analysis (HCA) of all Qingpi samples based on the color parameters was performed using Origin 2022 software (OriginLab Corporation, Northampton, MA, USA). The processing of the metabolomics data was carried out as previously described. Difference significance analysis was carried out using SPSS 20.0 software (SPSS Inc., Chicago, IL, USA), and *p* < 0.05 was considered indicative of a significant difference by one-way ANOVA analysis.

## 3. Results and Discussion

### 3.1. Color Change of Qingpi Samples during Storage

**Figure 1:**
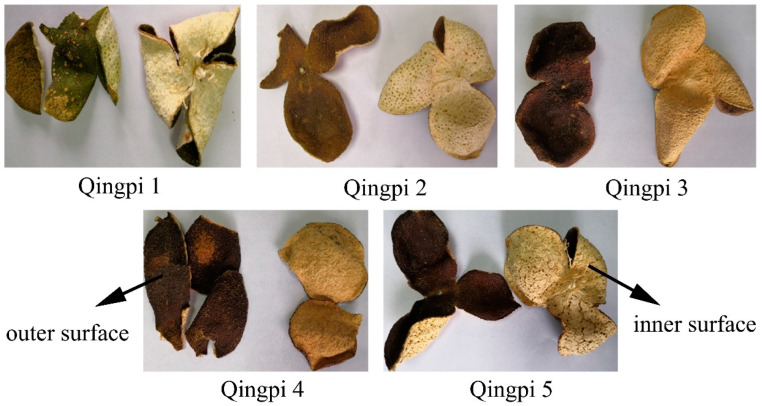
Colors of Qingpi 1, Qingpi 2, Qingpi 3, Qingpi 4, and Qingpi 5 samples during 1, 2, 3, 4, and 5 years of storage, respectively.

**Figure 2:**
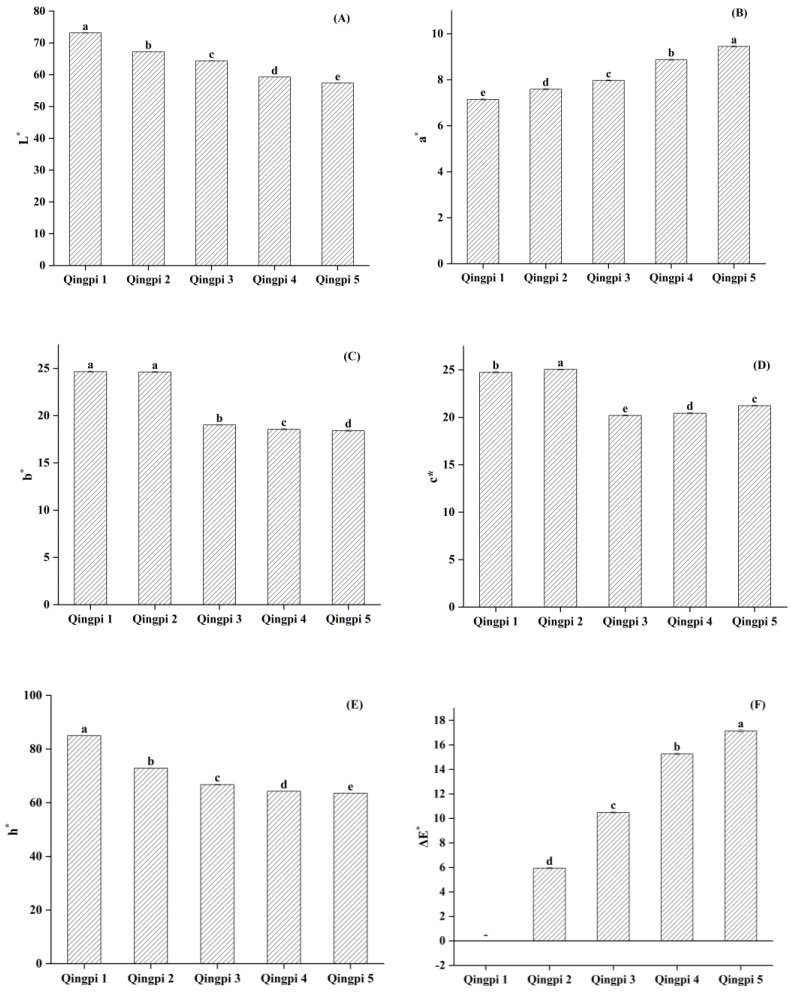
The color parameters (**A**–**F**: L*, a*, b*, c*, h*, and ΔE*) of Qingpi 1, Qingpi 2, Qingpi 3, Qingpi 4, and Qingpi 5 samples during 1, 2, 3, 4, and 5 years of storage, respectively. Different letters indicate significant differences at *p* < 0.05. “−” means that all ΔE* values of Qingpi are calculated based on this datum in (**F**).

Color was the most intuitive change in Qingpi appearance during storage. As shown in Figure 1, the color of the outer and inner surfaces of Qingpi samples gradually changed from dark green to dark brown and from yellowish-white to yellowish-brown, respectively, with prolonged storage. Due to the influence of human subjectivity, the naked eye has the disadvantage of accuracy in observing color change [31]. Therefore, a color difference meter was used to determine the powder color of the Qingpi samples. In this process, the color can be quantified as specific values, which overcomes the deficiency of naked-eye measurements. The determination results of the Qingpi samples during 1 to 5 years of storage are shown in Figure 2.

As shown in Figure 2A–F, the L* value of Qingpi decreased significantly (*p* < 0.05) during storage, indicating the brightness darkened and the color deepened. The b* value of Qingpi also decreased significantly (*p* < 0.05), indicating that the yellow color weakened. The values of a* and ΔE* of Qingpi increased significantly (*p* < 0.05) along with the prolonging of storage time. Among them, ΔE* indicates the magnitude of the color difference [32], with a greater color difference represented by a lower value. In addition, the c* and h* values of Qingpi also significantly decreased (*p* < 0.05). These results indicated that the color of the Qingpi samples gradually deepened during storage, in accordance with those of Chenpi and dried lemon slices during storage [23,32].

**Figure 3:**
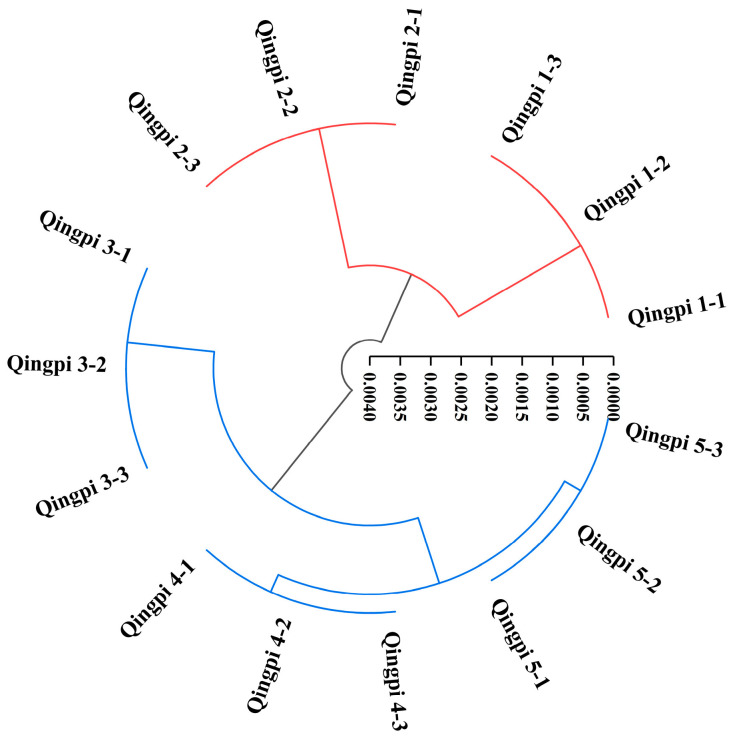
Hierarchical cluster analysis of Qingpi 1, Qingpi 2, Qingpi 3, Qingpi 4, and Qingpi 5 samples during 1, 2, 3, 4, and 5 years of storage, respectively, based on the color parameters.

HCA provides a way to visualize the hidden relationships between investigated samples and presents a clustering pattern of investigated elements [33]. Based on the color parameters, the cluster analysis of Qingpi samples during 1 to 5 years of storage was performed, as shown in Figure 3. The Qingpi samples were divided into two categories, Qingpi 1 to Qingpi 2 and Qingpi 3 to Qingpi 5, which indicated that the color of Qingpi stored for the first 2 years was similar, and that stored for 3 years or more was also similar, but there was obviously a difference between the sets of samples. Our field investigation found that farmers in Xinhui District usually stored Qingpi for a period of time before sale to preserve a deeper color and obtain better quality. However, there are few reports on the storage process of Qingpi, and the number of storage years resulting in better quality Qingpi was not also supported by theoretical data. Therefore, levels of the main flavonoids, TFs, and TPs, and the antioxidant activity were determined to further study the quality changes of Qingpi during storage.

### 3.2. HPLC Method Validation

In the study, the HPLC-DAD method was used to determine the levels of hesperidin, nobiletin, and tangeretin in Qingpi. The calibration curves were plotted with six concentrations of standard solutions. Acceptable linear correlation at those conditions was confirmed by the correlation coefficients (*R*^2^ ≥ 0.9998), as shown in Table 1. The typical HPLC chromatograms of both the mixed standard solution and sample solutions are shown in Figure S1.

The relative standard deviation (RSD) values of hesperidin, nobiletin, and tangeretin were 0.15%, 0.27%, and 0.21%, respectively, indicating that the method had high precision. The RSD values of hesperidin, nobiletin, and tangeretin were 2.46%, 0.93%, and 0.91%, respectively, which revealed that the method had high repeatability. The RSD values of hesperidin, nobiletin, and tangeretin were all less 3%, indicating that the sample solution remained stable within 48 h. The accuracy of the method was further evaluated via the recovery test. The mean recovery rates of hesperidin, nobiletin, and tangeretin were 97.43%, 99.82%, and 100.16% (all RSD ≤ 2.81), respectively (Table S1).

### 3.3. Analysis of Flavonoids of Qingpi Samples during Storage

Flavonoids are the main active compounds in Qingpi, and the hesperidin content of Qingpi was reported in the Chinese Pharmacopoeia (2022 edition) [3]. Previous reports also identified polymethoxylated flavones (such as nobiletin and tangeretin) as the major flavonoids in Qingpi [2,4]. Therefore, the levels of three flavonoids (hesperidin, nobiletin, and tangeretin) were determined in Qingpi and the determination results are shown in Table 2.

As shown in Table 2, the hesperidin content decreased from 46.65 ± 0.36 to 27.49 ± 0.14 mg/g (*p* < 0.05) in Qingpi during 1 to 5 years of storage. This result was consistent with the decreasing trend in Chenpi during storage [34]. The levels of nobiletin and tangeretin decreased from 6.60 ± 0.04 to 4.21 ± 0.02 mg/g (*p* < 0.05) and from 4.70 ± 0.03 to 2.58 ± 0.02 mg/g (*p* < 0.05), respectively. Different from our results, the levels of nobiletin and tangeretin increased in Chenpi during 1 to 11 years of storage [23,34]. Qingpi has the same origin as Chenpi in family, genus, species, producing area, and medicinal part, but Chenpi is the mature pericarp whereas Qingpi is the immature pericarp. At present, the accepted view of the aging mechanism of Chenpi is “the longer the storage, the better the quality” owing to the increased levels of nobiletin and tangeretin with prolonged storage [23]. Obviously, Qingpi does not fit the aging mechanism of Chenpi.

### 3.4. Changes in TFs, TPs, and Antioxidant Activity of Qingpi during Storage

The TP content is closely related to the antioxidant capacity [23,35,36]. As shown in Table 2, there was no significant difference in TP content in Qingpi stored for one, two, and five years (*p* > 0.05) and the difference was also not significant between the third and fourth year of storage (*p* > 0.05). However, the TP content in Qingpi stored for three years was significantly lower than that stored for one and two years (*p* < 0.05). There were two directions for the fluctuation in TP content during the storage of Qingpi. The decreasing direction was due to cell structure destruction and non-enzymatic oxidation occurring during storage [23,37,38]. The increasing direction was due to the relative instability of some flavonoids, which are prone to multiple reactions and modifications during storage [39]. The TF content was stable for 1 to 2 years of storage and was significantly different from that of 3 to 5 years of storage (*p* < 0.05). The TF content showed a decreasing trend with prolonged storage of Qingpi, which was also observed in Chenpi and broccoli during storage [23,40].

At present, some analytical methods, such as DPPH, FRAP, the 2,2′-azinobis (3-ethylbenzo thiazoline-6-sulfonic acid) diammonium salt (ABTS) assay, and the photochemiluminescence assay, in both water-soluble (ACW) and lipid-soluble (ACL) modes, have been applied to determine the antioxidant capacity of antioxidant compounds [23,35]. However, the antioxidant activity of plants can be caused by different mechanisms, including scavenging of free radicals, decomposition of peroxide, and prevention of chain initiation. Meanwhile, plant composition is complex, and using only one method limits the determination of antioxidant activity of plant materials [23,41]. Therefore, both the DPPH and FRAP assays were used to evaluate the antioxidant activity of Qingpi samples during storage.

As shown in Table 2, the DPPH values of Qingpi samples displayed a significant downward trend in the first three years of storage (*p* < 0.05) and a stable trend in the fourth and fifth years of storage (*p >* 0.05). The FRAP values of Qingpi samples stored for 1 to 2 years had no significant changes (*p >* 0.05), but were significantly higher than that of samples stored for three years or more (*p* < 0.05). Many studies have reported that the antioxidant activity of some medicinal plants, vegetables, and fruits generally decrease with prolonged storage time [42,43,44], which was in agreement with our results. The decreasing trend could be related to the oxidation of antioxidant compounds over time.

### 3.5. LC-MS Untargeted Metabolomics Analysis Results of Qingpi

**Figure 4:**
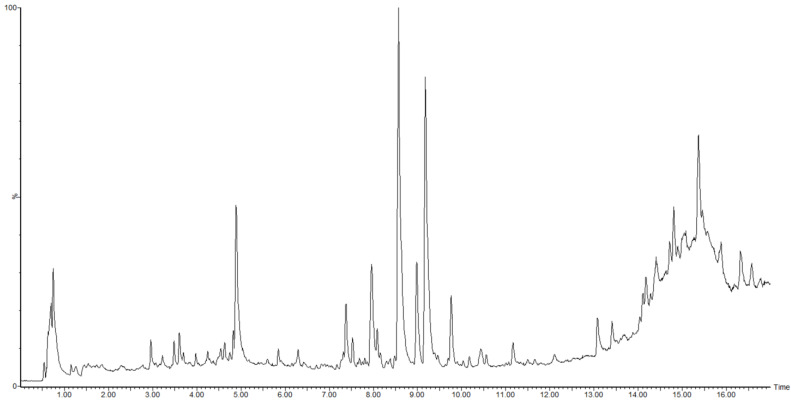
LC-MS total ion chromatogram of Qingpi 4 sample.

The plant metabolomics information of all Qingpi samples was determined by UPLC-Q/TOF-MS. The total ion chromatogram of the Qingpi 4 sample is shown in Figure 4. The MS data were obtained by Progenesis QI software and used for multivariate statistical analysis.

**Figure 5:**
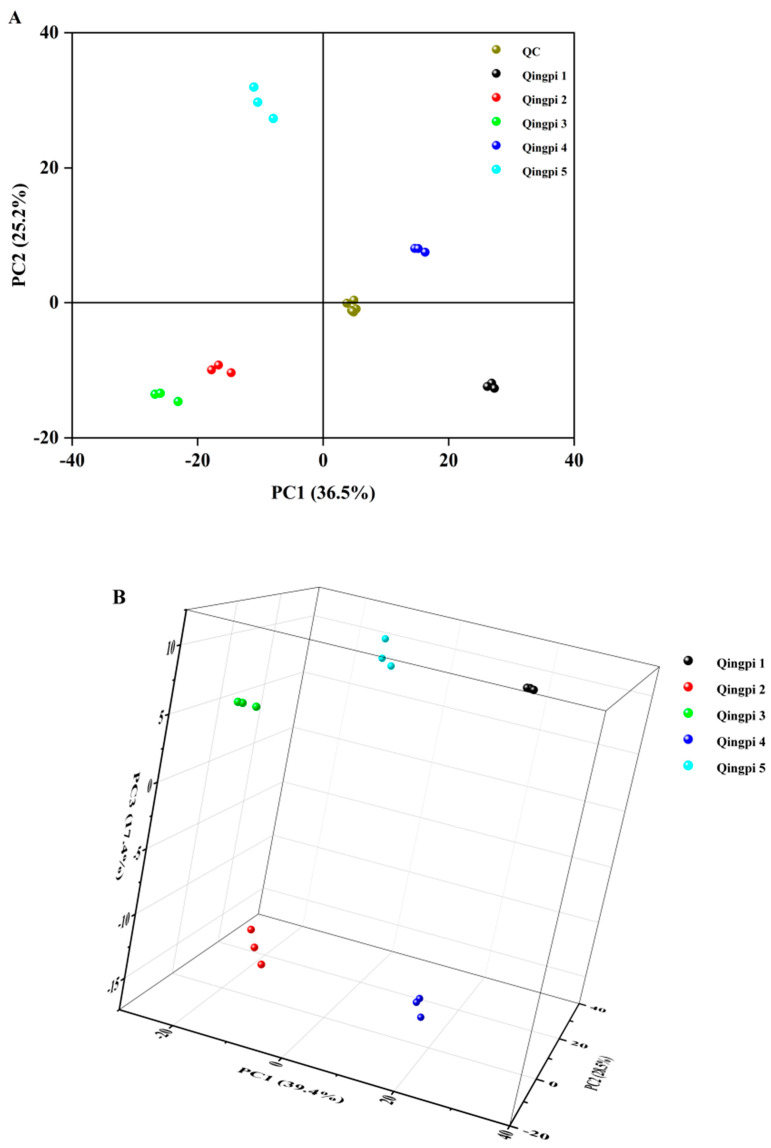
Multivariate analysis of LC-MS−based metabolomics data of Qingpi 1, Qingpi 2, Qingpi 3, Qingpi 4, and Qingpi 5 samples stored for 1, 2, 3, 4, and 5 years, respectively. Two−dimensional score plots of principle component analysis (PCA) with QC samples (**A**). Three-dimensional score plots of PCA without QC samples (**B**).

In the process of plant metabolomics analysis, the verification of data quality is an important step and requires high-quality and reliable data results from QC samples [45]. As an unsupervised pattern recognition method, PCA can reveal the interrelationships between different variables and interpret sample patterns, groupings, similarities, and differences [30]. The two−dimensional PCA scores plot of Qingpi samples during 1 to 5 years of storage is shown in Figure 5A. All QC samples were distributed near the origin of coordinates and clustered together, which confirmed the system was stable. In addition, it was intuitively discovered that samples of the same storage year were close to each other in distance, indicating little difference within a group, while the distance between samples of different storage years was great, suggesting obvious differences among groups. Interestingly, the Qingpi samples stored for 1 to 3 years and 4 to 5 years were distributed on the negative and positive half axes of the vertical axis, respectively, indicating that the metabolites of Qingpi before and after 3 years of storage had significant differences. The three-dimensional PCA scores plot (without QC samples) is shown in Figure 5B. The total values of PC1 (*X*-axis), PC2 (*Y*-axis), and PC3 (*Z*-axis) accounted for 85.3% of the total variance. As shown in Figure 5B, the samples within one group were clustered together in space, and no overlap occurred among groups. These results indicated that the PCA method could well distinguish Qingpi samples with different storage years.

As a supervised stoichiometric approach, the PLS-DA model focuses on differences among samples from different classes [46]. In order to obtain the VIP values for screening differential metabolites of Qingpi stored for 1 to 5 years, the PLS-DA model (*R*^2^*X* = 0.977, *R*^2^*Y* = 0.991, and *Q*^2^ = 0.983) was established. After 200 permutation tests, the intersection point between the *Q*^2^ regression line and vertical axis was less than zero, indicating that there was no over-fitting in the model [22].

### 3.6. Screening and Identifying of Special Differential Metabolites of Qingpi

**Figure 6:**
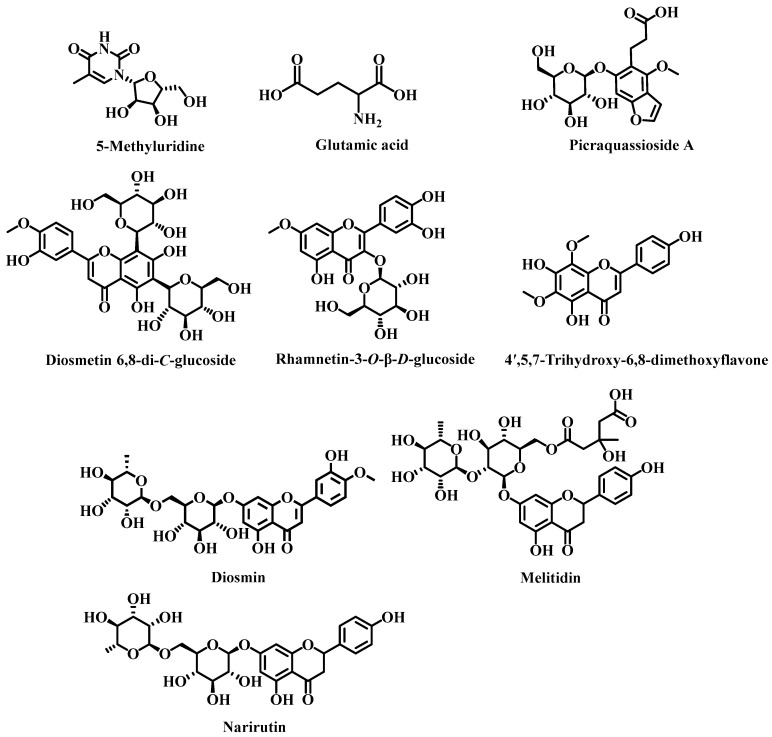
Chemical structures of 9 special differential metabolites.

The VIP value in the PLS-DA model represents the contribution of each compound to the classification [34]. The larger the VIP value, the more significant the difference of compound. Values of VIP > 1 can be used to assist the screening of differential metabolites [47]. In the present study, VIP > 2 was used in the process of analysis to obtain special differential metabolites. Hence, according to the conditions of VIP > 2, ANOVA *p* < 0.05, and max fold change > 1.5, a total of 21 chromatographic peaks were screened out. Among them, 9 special differential metabolites (as shown in Table 3) were finally identified by checking standards and referencing the literature and databases [48,49,50,51,52,53,54]. Their chemical structures are shown in Figure 6. The retrieved databases consisted of ChemicalBook (https://www.chemicalbook.com/ProductIndex.aspx, accessed on 2 November 2021), ChemSpider (http://www.chemspider.com/, accessed on 2 November 2021), and PubChem (https://pubchem.ncbi.nlm.nih.gov/, accessed on 3 November 2021). For example, compound No. 5 (retention time of 4.62 min) was identified as narirutin based on the primary and secondary mass spectrograms of the ion peak by comparing against the standard substance, as shown in Figure S2A–D.

**Figure 7:**
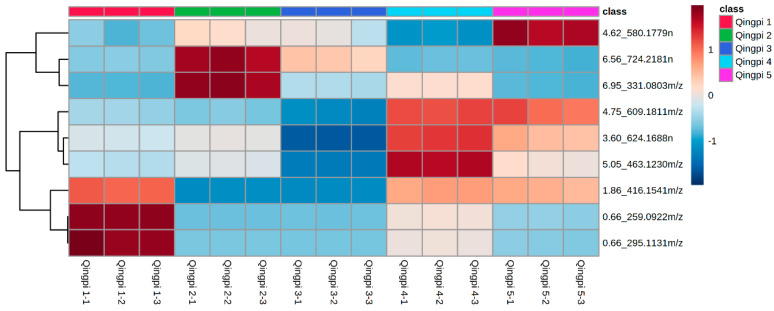
Heat map of 9 special differential metabolites in Qingpi 1, Qingpi 2, Qingpi 3, Qingpi 4, and Qingpi 5 samples during 1, 2, 3, 4, and 5 years of storage, respectively.

A heat map was plotted based on the MS data of the 9 special differential metabolites. The different levels of these metabolites can be intuitively and clearly observed in Figure 7. The levels of 5-methyluridine, glutamic acid, and picraquassioside A in Qingpi were higher in the first year of storage, and the levels of melitidin and 4′,5,7-trihydroxy-6,8-dimethoxyflavone were higher in the second year of storage. In addition, levels of narirutin, diosmin, diosmetin 6,8-di-*C*-glucoside, and rhamnetin-3-*O*-β-*D*-glucoside were higher in the fourth and fifth years of storage. These results indicated that 9 special differential metabolites could be used to distinguish Qingpi samples with different storage years.

Among the 9 special differential metabolites, 6 compounds were flavonoids. Interestingly, three flavonoids (hesperidin, nobiletin, and tangeretin), with their content previously determined, did not belong to the special differential metabolites. The reason may be that the screening of special differential metabolites must simultaneously meet three conditions described. Among them, narirutin is a dihydroxy flavanone glycoside with some anti-cancer, neuroprotective, stress-relieving, liver-protecting, anti-allergic, anti-diabetic, anti-obesity, antioxidant, anti-inflammatory, and immunomodulatory effects [55]. Diosmetin 6,8-di-*C*-glucoside is a flavonoid glycoside with some anti-oxidation, anti-aging [56], and anti-edema effects [49]. Diosmin is a flavanone glycoside with chondro-protective effects for human articular chondrocytes under oxidative stress [57], potential protective effects against streptozotocin-induced diabetic cardiomyopathy in rats [58], and ameliorative effects against doxorubicin-induced nephrotoxicity [59]. Rhamnetin-3-*O*-β-*D*-glucoside is also a common flavanone glycoside with antimelanoma and antioxidant activities [60,61]. Glutamic acid participates in many important biochemical reactions [62] and 5-methyluridine is involved in plant growth, so their gradually decreasing levels indicated that the life process of Qingpi was gradually weakened during storage.

## 4. Conclusions

During 1 to 5 years of storage, deeper color was the most intuitive change in Qingpi, reflected by changes in color parameters with L*, b*, c*, and h* values decreasing and a* and ΔE* values increasing. The color changes of Qingpi during storage were summarized. Browning is a common phenomenon in the storage of foods and natural products, generally accompanied by a deepening of color [23,63]. Qingpi and Chenpi are the same in origin, drying processing, and storage conditions. It had been confirmed that non-enzymatic browning is the reason for the deeper color of Chenpi during storage [28]. Therefore, the same conclusion can be drawn that the deeper color of Qingpi during storage is also caused by non-enzymatic browning. This is the first study to elucidate the phenomenon and mechanism of color change in Qingpi during storage. The cluster analysis performed based on the color parameters showed that the Qingpi samples were divided into two categories, one for the first and second year of storage and another for the third, fourth, and fifth year of storage. During 1 to 5 years of storage, levels of flavonoids (hesperidin, nobiletin, and tangeretin), TFs, and antioxidant activity showed a downward trend in Qingpi, especially during 1 to 3 years of storage (*p* < 0.05).

Based on these phenomena, LC-MS untargeted metabolomics analysis combined with multivariate statistical methods were used to identify the metabolite changes in Qingpi during storage. The PCA results indicated that the metabolites of samples before and after 3 years of storage had significant differences. A total of 21 chromatographic peaks were screened out, which simultaneously met the three conditions including VIP > 2, ANOVA *p* < 0.05, and max fold change > 1.5. Among the 21 chromatographic peaks, 9 special differential metabolites were identified. The heat map showed that levels of 5-methyluridine, glutamic acid, and picraquassioside A were highest in the first year of storage, levels of melitidin and 4′,5,7-trihydroxy-6,8-dimethoxyflavone were highest in the second year, and levels of narirutin, diosmin, diosmetin 6,8-di-*C*-glucoside, and rhamnetin-3-*O*-β-*D*-glucoside were highest in the fourth and fifth years. Therefore, the identified 9 special differential metabolites could be used to distinguish Qingpi samples with different storage years. However, the highest levels of the special differential metabolites were not found in the third year of storage. This is the first study to report the differential metabolites of Qingpi with different storage years analyzed using untargeted metabolomics.

In summary, based on the evaluation and optimization results of quality, including physicochemical characteristics and metabolite changes, we found that color deepening, decreasing and increasing levels of some compounds, decreasing antioxidant activity, and changes in differential metabolites occurred in Qingpi during storage. Therefore, it is suggested that medicinal and edible Qingpi should be stored for no more than 3 years to ensure the best quality. Furthermore, the temperature and relative humidity during storage should be strictly controlled to prevent the production of harmful microorganisms.

## Figures and Tables

**Table 1:** HPLC calibration curves for hesperidin, nobiletin, and tangeretin.

Compound	Linear Range (mg/mL)	Linear Equation ^1^	Correlation Coefficient (*R*^2^)
Hesperidin	0.0075–0.24	*y* = 16480313*x* + 14093	0.9998
Nobiletin	0.0025–0.08	*y* = 36366264*x* + 12348	0.9999
Tangeretin	0.0025–0.08	*y* = 40035978*x* + 13531	0.9999

^1^ *y* and *x* express the peak area and corresponding injection concentration (mg/mL), respectively.

**Table 2:** Levels of hesperidin, nobiletin, tangeretin, TFs, and TPs and the antioxidant activity of Qingpi during storage (mean ± SD, *n* = 3).

Sample	Hesperidin (mg/g)	Nobiletin (mg/g)	Tangeretin (mg/g)	TFs (mg CE/g DW)	TPs (mg GAE/g DW)	FRAP (μM TE/g DW)	DPPH (μM TE/g DW)
Qingpi 1	46.65 ± 0.36a	6.60 ± 0.04a	4.70 ± 0.03a	3.90 ± 0.24a	19.14 ± 1.32a	79.80 ± 0.79a	25.30 ± 0.20a
Qingpi 2	35.00 ± 0.06b	5.38 ± 0.03b	4.00 ± 0.02b	3.88 ± 0.11a	19.20 ± 1.11a	79.60 ± 0.96a	24.16 ± 0.13b
Qingpi 3	34.50 ± 0.17c	4.66 ± 0.04c	3.34 ± 0.02c	2.66 ± 0.26b	17.42 ± 0.37b	77.60 ± 0.72b	22.08 ± 0.25c
Qingpi 4	30.32 ± 0.10d	4.21 ± 0.02d	3.06 ± 0.03d	2.96 ± 0.14b	18.80 ± 0.71ab	76.88 ± 0.97b	21.90 ± 0.89c
Qingpi 5	27.49 ± 0.14e	4.23 ± 0.04e	2.58 ± 0.02e	2.84 ± 0.25b	20.19 ± 0.70a	76.67 ± 0.73b	21.75 ± 0.31c

The different letters in the same column indicate significant differences at *p* < 0.05.

**Table 3:** Special differential metabolites screened from Qingpi samples stored for 1 to 5 years.

No.	Rt (min)	Compound	MF	MW	VIP	Max Fold Change
1	0.66	5-Methyluridine	C_10_H_14_N_2_O_6_	259.0922 *m*/*z*	3.83	11.97
2	0.66	Glutamic acid	C_5_H_9_NO_4_	295.1131 *m*/*z*	3.01	7.43
3	1.86	Picraquassioside A	C_18_H_22_O_10_	416.1541 *m*/*z*	2.19	9.32
4	3.60	Diosmetin 6,8-di-*C*-glucoside	C_28_H_32_O_16_	624.1688 *n*	5.04	3.84
5	4.62	Narirutin *	C_27_H_32_O_14_	580.1779 *n*	2.71	1.62
6	4.75	Diosmin	C_28_H_32_O_15_	609.1811 *m*/*z*	2.59	1.67
7	5.05	Rhamnetin-3-*O*-β-*D*-glucoside	C_22_H_22_O_12_	463.1230 *m*/*z*	2.27	6.26
8	6.56	Melitidin	C_33_H_40_O_18_	724.2181 *n*	2.42	2.45
9	6.95	4′,5,7-Trihydroxy-6,8-dimethoxyflavone	C_17_H_14_O_7_	331.0803 *m*/*z*	2.69	4.59

“Rt” retention time, “MF” molecular formula, “MW” molecular weight. “VIP” is obtained from the PLS-DA model, “Max fold change” is obtained by Progenesis QI software, “*n*” represents the neutral molecular weight calculated by various adduct methods, “*m*/*z*” refers the mass-to-charge ratio of ion peak, * indicates the compound was identified according to the reference substance.

## Data Availability

Data is contained within the article.

## Supplementary Materials

The following supporting information can be downloaded at: https://www.mdpi.com/article/10.3390/foods12030463/s1, Figure S1: Typical HPLC chromatograms of mixed standard solution (A and B) and samples (Qingpi 1) (C and D). Numbers 1, 2, and 3 denote hesperidin, nobiletin, and tangeretin, respectively; Figure S2: Mass spectra of reference substance (narirutin) and sample. Primary (A) and secondary (B) mass spectra of narirutin; Primary (C) and secondary (D) mass spectra of peak at 4.62 min retention time of the sample; Table S1: Recovery tests of hesperidin, nobiletin, and tangeretin in Qingpi sample (Qingpi 1).
