# Supplementary material for: Evaluation and Optimization of Quality Based on the Physicochemical Characteristics and Metabolites Changes of Qingpi during Storage
Source: Foods. 2023 Jan 19;12(3):463. doi: 10.3390/foods12030463 (PMC9914837; doi:10.3390/foods12030463)
Supplement: Supplementary file 1 [file foods-12-00463-s001.zip › foods-2118306-supplementary.pdf]

Table S1. Recovery tests of hesperidin, nobiletin, and tangeretin in Qingpi sample (Qingpi 1).

Compounds	Sample (g)	Original (mg)	Addition (mg)	Detected (mg)	Recovery (%)	Average recovery (%)	RSD (%)
Hesperidin	0.2516	2.966	3.01	5.839	95.44	97.43	1.75
	0.2521	2.973	3.06	5.943	97.07		
	0.2510	2.960	3.03	5.860	95.69		
	0.2510	2.960	3.10	5.994	97.88		
	0.2517	2.967	3.13	6.057	98.70		
	0.2512	2.962	3.11	6.065	99.78		
Nobiletin	0.2516	1.427	1.42	2.855	100.56	99.82	2.81
	0.2521	1.430	1.40	2.861	102.23		
	0.2510	1.424	1.44	2.828	97.52		
	0.2510	1.408	1.49	2.8290	95.35		
	0.2517	1.396	1.45	2.861	100.97		
	0.2512	1.379	1.38	2.790	102.31		
Tangeretin	0.2516	1.000	1.01	2.045	103.44	100.16	2.59
	0.2521	1.002	1.10	2.096	99.40		
	0.2510	0.9981	1.11	2.091	98.47		
	0.2510	0.9872	1.07	2.042	98.63		
	0.2517	0.9789	1.03	2.044	`103.42		
	0.2512	0.9664	0.98	1.923	97.61		

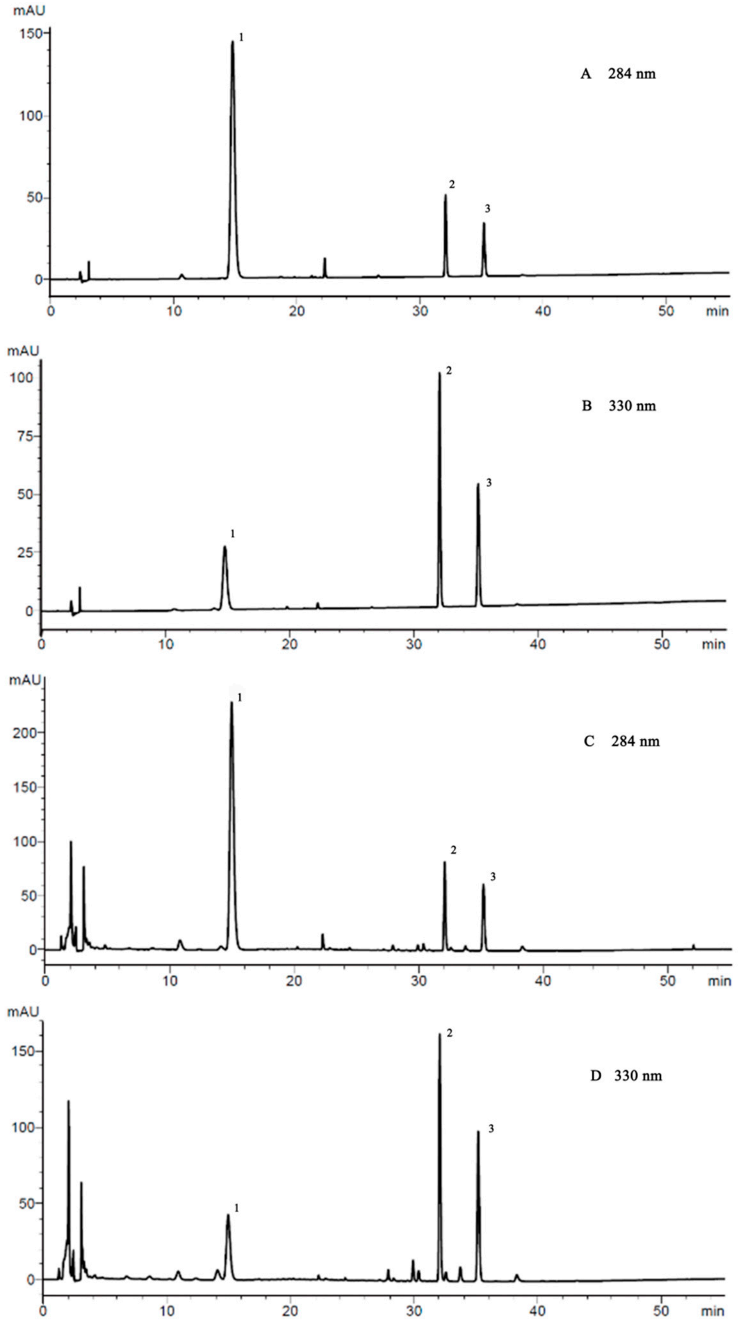

Figure S1. Typical HPLC chromatograms of mixed standard solution (A and B) and samples (Qingpi 1) (C and D).

Numbers 1, 2, and 3 denote hesperidin, nobiletin, and tangeretin, respectively.

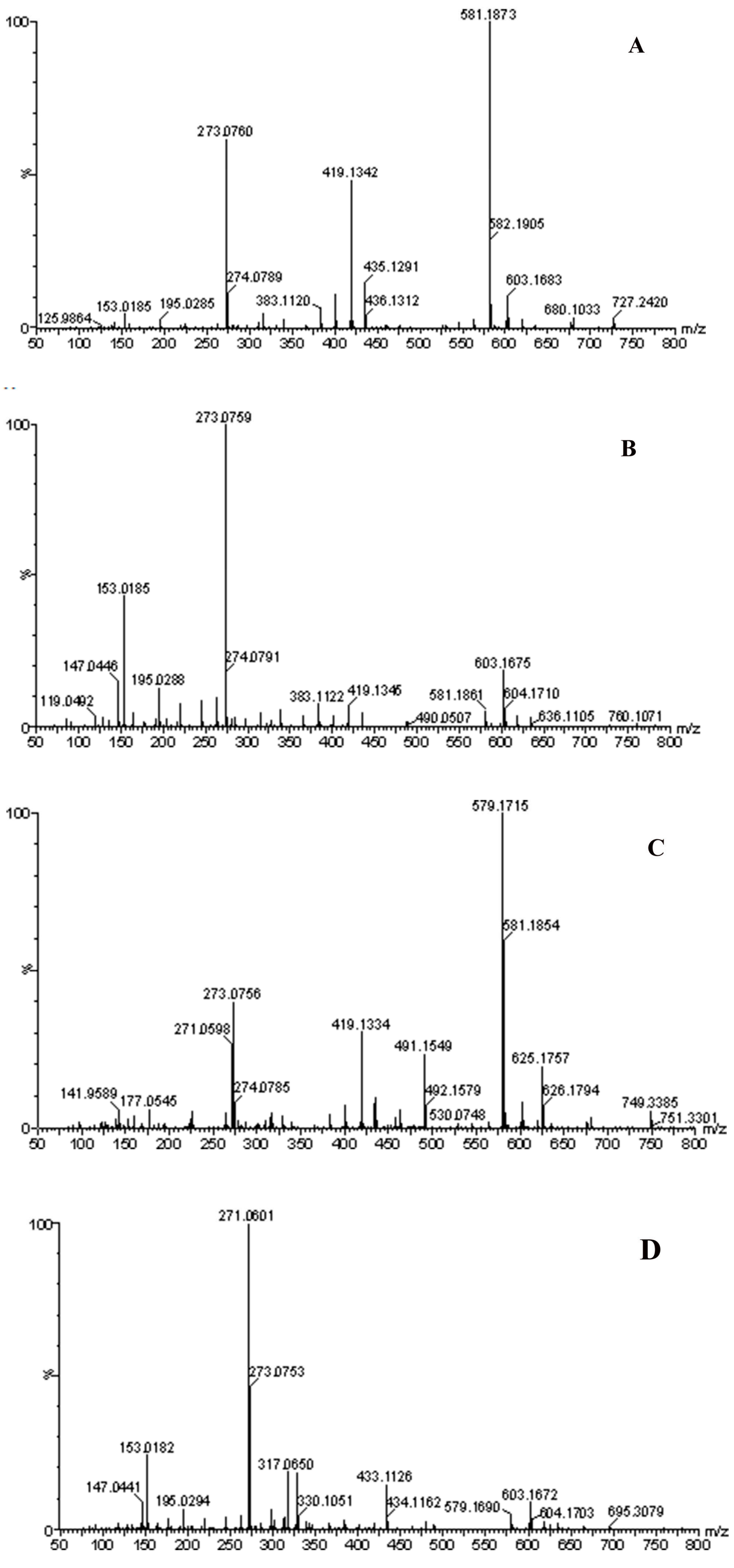

Figure S2. Mass spectra of reference substance (narirutin) and sample. Primary (A) and secondary (B) mass spectra of narirutin; Primary (C) and secondary (D) mass spectra of peak at 4.62 min retention time of the sample.
